# An Improved Encoder-Decoder Network Based on Strip Pool Method Applied to Segmentation of Farmland Vacancy Field

**DOI:** 10.3390/e23040435

**Published:** 2021-04-08

**Authors:** Xixin Zhang, Yuhang Yang, Zhiyong Li, Xin Ning, Yilang Qin, Weiwei Cai

**Affiliations:** 1 College of Information Engineering, Sichuan Agricultural University, Ya’an 625000, Sichuan, China; zhangxixin@stu.sicau.edu.cn (X.Z.); yangyuhang@stu.sicau.edu.cn (Y.Y.); 2 Sichuan Key Laboratory of Agricultural Information Engineering, Ya’an 625000, Sichuan, China; 3 Institute of Semiconductors, Chinese Academy of Sciences, Beijing 100083, China; ningxin@semi.ac.cn; 4 Institute of Agricultural Economy and Information, Henan Academy of Agricultural Sciences, Zhengzhou 450002, Henan, China; indexlang@outlook.com; 5 College of Logistics and Transportation, Central South University of Forestry and Technology, Changsha 410004, Hunan, China; vivitsai@ieee.org

**Keywords:** semantic segmentation, farmland vacancy segmentation, strip pooling, crop growth assessment, encoder–decoder

## Abstract

In the research of green vegetation coverage in the field of remote sensing image segmentation, crop planting area is often obtained by semantic segmentation of images taken from high altitude. This method can be used to obtain the rate of cultivated land in a region (such as a country), but it does not reflect the real situation of a particular farmland. Therefore, this paper takes low-altitude images of farmland to build a dataset. After comparing several mainstream semantic segmentation algorithms, a new method that is more suitable for farmland vacancy segmentation is proposed. Additionally, the Strip Pooling module (SPM) and the Mixed Pooling module (MPM), with strip pooling as their core, are designed and fused into the semantic segmentation network structure to better extract the vacancy features. Considering the high cost of manual data annotation, this paper uses an improved ResNet network as the backbone of signal transmission, and meanwhile uses data augmentation to improve the performance and robustness of the model. As a result, the accuracy of the proposed method in the test set is 95.6%, mIoU is 77.6%, and the error rate is 7%. Compared to the existing model, the mIoU value is improved by nearly 4%, reaching the level of practical application.

## 1. Introduction

Since a few years ago, the segmentation of farmland based on remote sensing images has been studied extensively owing to the development of deep learning technology, making intelligent farmland a hot trend. Based on their shooting distance, satellites and unmanned aerial vehicles are two common photographing tools. Satellite-based remote sensing images are mainly used in the segmentation of farmland forest areas such as Han Lin Aung, Burak Uzkent, etc., using space–time convolution networks to circle the farmland plots [1]. Guang Wang, Fan Yu, and others used the segmentation models of farmland and woodland with the help of remote sensing images to segment the farmland and woodland [2]. Ganchenko et al. used convolutional neural networks based on agricultural vegetation monitoring image semantic segmentation [3]. Drone-based remote sensing images are mainly used in specific scene analysis of a field, such as Yang’s [4] deep semantic segmentation techniques, Fully Convolutional Networks (FCN), SegNet, etc., which segment the farmland covered by plastic mulch to study environmental and soil contamination. There are crop and weed segmentations studied by Lei F. Tian and Heping Zhu et al. [5] using drones to capture low-altitude images of crops for crop morphological segmentation [6].

Wheat is the world’s largest ration crop; it has high economic, medicinal, and nutritional value, according to the Journal of Experimental Botany [7]. Therefore, we take wheat crops as an example to study.

The satellite image monitoring used previously is a very macro means of observation. Many problems are often faced in the early stages of wheat cultivation. Many factors can make wheat crops grow poorly in the early stages. The main factors leading to their initial dysplasia can be classified as environmental factors and their own factors. Environmental factors mainly include weather, pests and diseases, soil, and other such external factors. In the previous application, the use of semantic segmentation techniques for early development monitoring of wheat included wheat insect identification [8] and wheat field weed segmentation [5], combined with sensor equipment to measure soil acidity and alkalinity, detect nutrients, monitor weather [9], and so on. During the study, it was found that a small proportion of wheat planted in the field could be stunted or lodged, or even directly died due to these influencing factors. This leads to a common phenomenon: the creation of vacancies in farmland. This performance is a direct reflection and manifestation of the poor growth of wheat. Therefore, we propose a method of segmenting wheat vacancies in the early stages, as it is an important factor in the growth of wheat, making it easy to consider in estimating yield.

This paper mainly studies the problems prevailing in the application of existing semantic segmentation technologies in farmland vacancy segmentation, analyzes the advantages and disadvantages of each method according to the comparison of training process and experimental results, and proposes a new semantic segmentation network based on encoder–decoder architecture. At the same time, according to the characteristics of the dataset, the algorithm is improved adaptively. This paper is divided into six parts. The background section introduces several mainstream semantic segmentation techniques and summarizes the contribution of this paper, followed by the dataset section, and the methods used in this paper and the training process are described later. The validity of the new model is then proved by experimental results.

## 2. Related Work

The introduction mentions that semantic segmentation technology has developed rapidly in recent years, which makes its application scope gradually expand and gradually cover all areas of life. However, in the field of agricultural application, the research on farmland vacancy segmentation is rarely involved. Therefore, we first study the development process and basic principles of the semantic segmentation algorithm, explore the application of these technologies in similar scenes, and analyze their advantages and disadvantages. According to these algorithms, we redesign and improve the algorithms in order to get the optimal algorithm for the scene.

As we all know, the FCN model [10] opens a new path for semantic segmentation research and solves the semantic-level image segmentation, and the U-Net [11] has achieved significant results in the field of biomedical segmentation, which mainly solves the problem of small sample training to extract detailed texture features, such as using it for yellow embroidery surveillance in wheat fields [12] and retinal vessel segmentation [13]. Thus, the semantic segmentation algorithm has made a leap forward. Both algorithms originate from the Convolutional Neural Network.

The Convolutional Neural Network (CNN) gained popularity because AlexNet [14] had achieved a higher score than traditional methods in an ImageNet image recognition competition. However, as it defines every pixel, the relationship between pixels cannot be fully considered, and the lack of spatial unity leads to poor performance in semantic level segmentation. Thus, the Full Convolutional Network (FCN) [10] was born, which changed the entire connection layer in the last layer of the earlier classification network. It thus changed the previous result of the output one-dimensional probability vector and realized output characteristic graph. At the same time, a combined deconvolution, upsampling, and skip structure method was used to achieve end-to-end training results. Similarly, combined with deconvolution, the method of upper sampling and jump structure was used, and it achieved pioneering results of end-to-end training.

In order to reduce the cost of computing in the process of network training, researchers propose a dense network architecture called DenseNet [15]. DenseNets are built from dense block sand pooling operations, where each dense block is an iterative concatenation of previous feature maps. This structure extension is also applied to FCN to form FC-DenseNet [16]. It exploits the feature reuse by extending the more complex DenseNet architecture by skipping connections in the original FCNs, while avoiding feature overflow on the sampling path on the network. Later, in order to better apply the semantic segmentation algorithm to the segmentation of complex scenes, PSPNet [17] was born. PSPNet, with an atrous convolution of FCN as a baseline, focusing on the three aspects of mismatched relations, confusion categories, and inconspicuous classes, designed a hierarchical global priority containing information on different scales between different sub-regions, called the pyramid pooling module. The module incorporates four different pyramid-scale features using a feature chart, which is pooled with four sizes of pooled cores, resulting in four different scales of the feature map. To maintain the weight of the global feature, we use a 1 × 1 convolution layer to reduce the dimension. Then a bilinear interpolation method is used to restore the size of the feature map and concatenate four different levels of features, and finally, the global features of the pyramid pool are obtained.

The idea of dense connection is applied to atrous spatial pyramid pooling (ASPP) to increase the receptive field. DenseASPP [18] has revealed that atrous convolution becomes less and less effective and gradually loses the modeling ability as the dilation rate of ASPP increases. Therefore, it is very important to find a network structure that can encode multi-scale information while obtaining a large enough receiving domain. DenseASPP combines the advantages of parallel and cascading using atrous convolution to map multi-scale features on a larger scale. Through a series of feature connections, neurons on each intermediate feature map encode semantic information from multiple scales, and different intermediate feature maps encode multi-scale information from different scales. Through a series of atrous convolutions, the neurons at later levels can obtain larger receptive fields, as there is no degeneration of the convolution kernel of ASPP. Thus, the final output of the DenseASPP model is a feature map that covers a wide range of semantic information in a very dense way.

DeepLabv3+ is an improved version of the DeepLab series [19,20,21]. After a series of developments, it is a mature algorithm for scene segmentation at present. DeepLabv3+ [22] added a decoder considering the problem of reduced feature map resolution and reduced prediction accuracy caused by the network layer with stride=2 in the ResNet network structure. The original DeepLabv3 as an encoder of the network structure, unlike the direct bilinear downsampling recovery feature map, has features that are first bilinearly upsampled by a factor of 4 and then concatenated with the corresponding low-level features from the network backbone that have the same spatial resolution. In addition, because of this improvement, the parameter volume increases. Here, Xception is used instead of ResNet as the backbone for training.

Through the development of these algorithms, we find that most researchers are improving their accuracy in some specific data sets, so that they can be applied to large scene analysis, automatic driving, and other fields. However, for some unique scenarios, these algorithms may not be able to show their advantages well. Of course, the development of these technologies also drives the research on the application of semantic segmentation algorithm in some specific scenes. Among them, the most similar to the research field of this paper is crack segmentation.

The rapid emergence of this technology has also led to the development of semantic segmentation in the field of crack segmentation. Mark David Jenkins, Thomas Arthur Carr [23], and others proposed a deep convolutional neural network for the semantic pixel-wise segmentation of road and pavement surface cracks, which also considered the cost of data acquisition and chose to conduct training in a small sample dataset. It finally achieved good results in a dataset with only 80 images. Similarly, Henrique Oliveira, Paulo Lobato Correia et al. [24] applied improved segmentation methods based on pixel refinement to pave the way for crack detection. Additionally, Martin Mayr, Mathis Hoffmann [25] used the improved ResNet50 network to segment the cracks in the EL image of the solar cell.

It is worth noting that farmland vacancy segmentation is more difficult as compared to cracks segmentation, because the vacancies are comprised of various sizes and shapes. Therefore, in most of the above-mentioned work, there are some drawbacks in the application of vacancy segmentation. Our work is mainly to improve the existing algorithm in this field to be suitable for the precise segmentation of crops and vacancies. Inspired by the DeepLabv3+ model [22], we used the encoder–decoder’s network architecture to better recover image information. At the same time, we leveraged a combination of strip pooling and spatial pyramid pooling to achieve a better segmentation of vacancies and crops. SPINet, the end-to-end method proposed by us, uses an improved ResNet network as an encoder and combines SPM and MPM modules to establish a self-attention mechanism. The decoder concatenates the output of the last layer of encoder and the output of the middle layer, and obtains the prediction graph by sampling up and recovering pixels.

Our contributions can be summarized as follows:

(1) We propose a semantic segmentation network, based on encoder–decoder architecture, which takes IResNet network as the backbone and integrates SPM and MPM modules to build the model self-attention mechanism.
(2) We take advantage of strip pooling to capture vacancies with greater precision.
(3) We provide a farmland dataset taken by Unmanned Aerial Vehicle, containing 320 pieces of training data, 80 pieces of test data, and 40 pieces of verification data.
(4) The model that we designed can adapt to the training of small samples and reach a good level.

## 3. Materials and Methods

### 3.1. Materials

The image data was collected in January 2019 at the Modern Agricultural Research and Development Base in Henan, China, covering an area of 948 acres. The filming site was a winter wheat test field of the Provincial Academy of Agricultural Sciences, and the experimental data were provided by the Agricultural Economy and Information Research of the Henan Academy of Agricultural Sciences. The shooting equipment used was DJI Phantom 4 Pro V2.0 Professional Intelligent 4K Ultra Clear Aerial Shooting Drone with shooting altitude of 20 meters. A total of 42 images were taken in the field, each one with more than 4000 × 4000 pixels. The pixels of each picture are too large, which is not conducive to training. Thus, we first cut out the excess areas of the image, and then cropped the image into four equal parts, by removing the edges of each image, ultimately obtaining 110 images, each of size 1024 × 1024. We randomly divided the data set into a training set, verification set, and test set according to the ratio of 8:1:2. In order to expand the training set, we cut the image size of 1024 × 1024 into four equal parts, and obtained 320 training images and 40 verification images. However, we used the size of 1024 × 1024 images for testing to better show the segmentation effect. Perez, Luis, and Jason Wang et al. [26] demonstrated the effectiveness of data enhancement in improving network performance through experimental comparison. A.Mikołajczyk and M.Grochowski et al. [27] describe some of the available methods of data augmentation and propose a better one. Therefore, we also use the data augmentation methods to improve the performance and robustness of the model.

**Figure 1:**
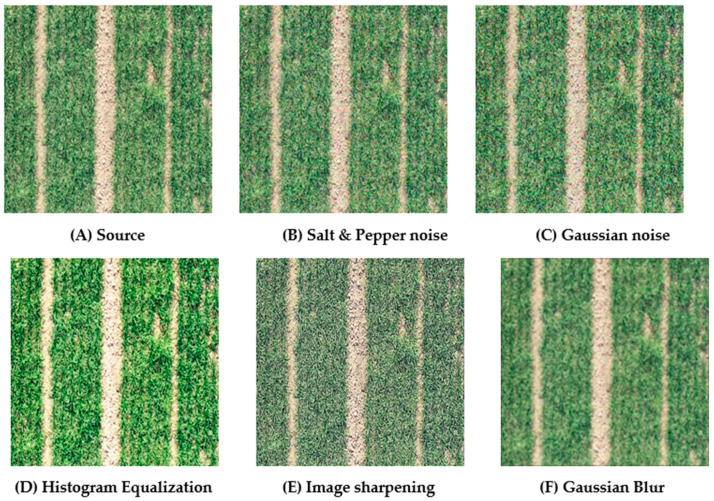
Five kinds of data augmentation operations for images. Explanation for subfigure (**A**) represents the original image, (**B**) represents the image after adding salt and pepper noise, (**C**) represents the image after adding Gaussian noise, (**D**) represents the image after histogram equalization, (**E**) represents the image after sharpening, and (**F**) represents the image after Gaussian blur.

The following Figure 1 shows the various actions we performed on the image.

It is worth noting that in this application field, crop category and crop vacancy category in the image need to be segmented. The vacancies in the wheat crop are many and very messy, as seen in the fields filmed, making the marking difficult and increasing the labeling time. The image acquisition cost is very high, and its marking requires the identification and judgment of experts, just like in the field of medical image segmentation. This, to some extent, aggravates the cost of using the model and requires researchers to design a practical model that can achieve good results in a small sample training set. Therefore, in this paper, considering the cost of data annotation, we design the small sample learning method. The algorithm improvement of small sample training will be explained later in detail. In this model, labelme was used for data labeling, which marked crops as red, vacancy as green, and backgrounds as black.

### 3.2. Methods

Based on the method proposed above (including the FC-DenseNet56, PSPNet, DenseASPP, and DeepLabv3+), we carried out a detailed comparative experiment and found that there exist certain disadvantages of the segmentation effect that need to be improved. By further analyzing the data characteristics and the training effect of each model on the dataset, a strip pool was re-selected to capture the characteristics of crop and vacancy, and the effect was much higher. In view of this, we redesigned a semantic segmentation method based on encoder–decoder architecture. In order to avoid the vacancy of other shapes that are difficult to catch in the strip pool, the MPM module, or mixed pool module, is built by combining PPM, which sets up different types of context information through various pool operations. Xin Ning et al. proposed a model combining weak saliency and attentional perception to obtain a more complete global feature by weakening the saliency feature [28]. This is another reason why we designed MPM.

Simultaneously, to improve the model’s predictive pixel accuracy for crops and the edge of the vacancy, a decoder module was added to recover more detailed information of the image, avoiding the loss of information by shrinking directly from 512-size channels to three channels. Finally, based on the particularity of little sample data training, inspired by Improve Residual Network, we have improved the signal transmission mode of the original ResNet to accommodate negative signal transmission during the initial training of low-sample training. This avoids eliminating too much information during transmission. Moreover, this paper also adopts IResNet as the backbone of architecture for the transmission characteristics of a few samples of training information, which changes the original means of signal transmission as compared to ResNet to preserve more useful information.

Below, we elaborate on our proposed methods.

#### 3.2.1. Encoder–Decoder Architecture

We designed a semantic split network with encoder–decoder architecture using IResNet, which combines SPM and MPM modules as a part of the encoder portion of the architecture and a decoder layer that combines the encoder layer output and the encoder middle layer output to sample and restore pixels. In most of the known segmentation methods, after sampling the features by a more granular convolution, it is often used directly to reduce the channels to three, i.e., revert to a visual 3D feature map. In case of such an operation, which can be understood as a minimalist decoder this is not conducive to the recovery of image features. Especially in our vacancy segmentation scene, there is a greater disadvantage in the segmentation of small vacancies as well as the segmentation of the edge of the vacancy. This arouses the need to design a more sophisticated decoder.

**Figure 2:**
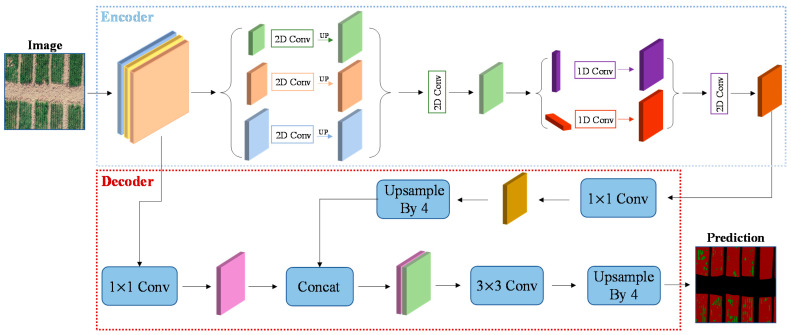
**Encoder–decoder network architecture:** The upper part of Figure 2 shows the encoder, taking IResNet as the main backbone, combining SPM and MPM modules. The lower part shows the decoder. It combines output of the last layer of encoder and the output connection of the middle layer to carry out up-sampling and restore feature map pixels. The first three-tier network in encoder represents IResNet + Strip Pooling Module (SPM) + Mixed Pooling Module (MPM). The arrows represent operations.

Figure 2 shows that the image input network structure begins with a series of convolutional normalization operations with IResNet backbone, and that in the process, there is a pool operation to extract feature of vacancy. It is important to note that the arrows in the figure do not refer to sequential operations but the directionality. Thus, it does not mean that the image enters MPM module before entering the SPM module. Additionally, the decoder can combine information of different scales. Therefore, we choose to combine the information of Layer2 and Layer4 in IResNet. Firstly, the channel of characteristic graph output by Layer4 in the encoder layer is converted into 512 by a 1 × 1 convolutional layer, and then sampled up by a factor of four connected to the output of Layer2 in the IResNet network (the output of Layer2 is also reduced by a 1 × 1 convolutional layer). Finally, the result is obtained by four-fold bilinear up-sampling recovery, after three 3 × 3 convolutional layers. To take into account the over-fitting of prevention model training, the output results of last layer of the network were not directly upsampled and recovered, and the contents of previous layers of the network were also combined. The comparison of model effects based on this architecture will be described in detail later, in the discussion section.

#### 3.2.2. SPM and MPM Modules

Strip pooling is a long, narrow pool kernel represented by 1 × N or N × 1. This pooling method helps to capture remote context information and establish a remote dependency, which can be seen as a self-attention mechanism. Compared to other mechanisms such as dilated convolutions and global/pyramidal pooling, the differences lie in the shape of the pooling nucleus and more suitable application scenes. A pyramid-like/pooling-like method is primarily a square window to detect input information to capture useful features. In this case, large receptive fields of the pooling kernel in the backbone network contribute to the scene analysis. The object characteristics of segmentation, in this case, are obvious and special, and it is more necessary to capture the features by using strip pooling. It is also known that a part of the vacancy tends to have long and narrow characteristics, consistent with the design thinking of strip pooling.

The difference between this pooling method and two-dimensional pooling is that strip pooling averages all feature values of rows or columns. As mentioned above, the spatial extent of pooling is 1 × N or N × 1. If given a two-dimensional vector $x\in R^{H\times W}$, the output $y^{h}\in R^{H}$ after horizontal strip pooling can be written as:

(1) $$\begin{array}{c} y_{i}^{h}=\frac{1}{W}\underset{0\leq j\leq W}{\sum }x_{i,j}. \end{array}$$

**Figure 3:**
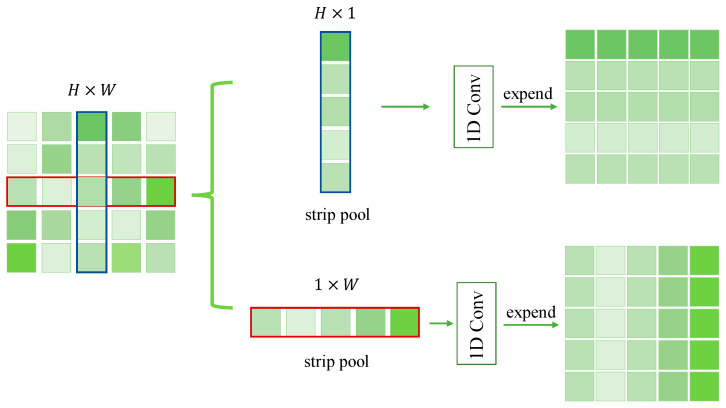
Schematic illustration of the strip pooling.

Similarly, the output $y^{v}\in R^{w}$ after vertical strip pooling can be written as:

(2) $$\begin{array}{c} y_{j}^{v}=\frac{1}{H}\underset{0\leq i\leq W}{\sum }x_{i,j}. \end{array}$$

where *H* and *W* are the spatial height and width, respectively. For this kind of pooling in the horizontal and vertical directions, it can build long-range dependencies to the remote information distributed in the horizontal and vertical directions, thus playing a role similar to the self-attention mechanism. It is also worth noting that because of its narrow kernel in a single direction, it is also more suitable for capturing local details. In Figure 3, we can see that the square-shape kernels are different from the previous one. At the same time, in the practical application process, it extends from horizontal and vertical directions, respectively, from H × 1 to H × W (or from 1 × W to H × W), and then through the fusion of the two (that is, to do the multiplication of elements) to do a further series of operations.

Based on this idea, two modules are constructed in this article, namely SPM and MPM. SPM contains 1 × N and N × 1 pool operations, followed by a 1D convolutional layer for modulating the current location and its neighboring features. Then, the two results are combined together. We then use a 1×1 convolution to reduce the number of channels of the feature graph and to increase the nonlinearity of the network. Later, ReLU layer normalization is used. Finally, the element-based multiplication is performed with the previous eigenmatrix. This module is primarily used to capture information about the long vacancy, and due to the characteristics of its kernel. As compared with the global average pooling operation, the number of parameters is less. The mixed pooling module (MPM) is designed primarily to address the vacancy of capturing various other shapes. As a result, the mixed pooling module is formed with a combination of standard pooling methods in PPM. As shown in Figure 2, after IResNet, three different sizes are formed through a standard pooling operation with three pooling kernel sizes, which are then fused. Another convolution layer is gone through to reduce the aliasing effect of the down-sampling operation. A new SPM module is entered, which traverses the input image with two strip pooling, 1 × N and N × 1, to capture features and scale horizontally and vertically to form a feature map, respectively. Additionally, another convolution layer should be gone through. In this case, the channel output of IResNet is reduced to 1024 from 2048 with a 1 × 1 convolutional layer before each submodule.

#### 3.2.3. Improved ResNet

**Figure 4:**
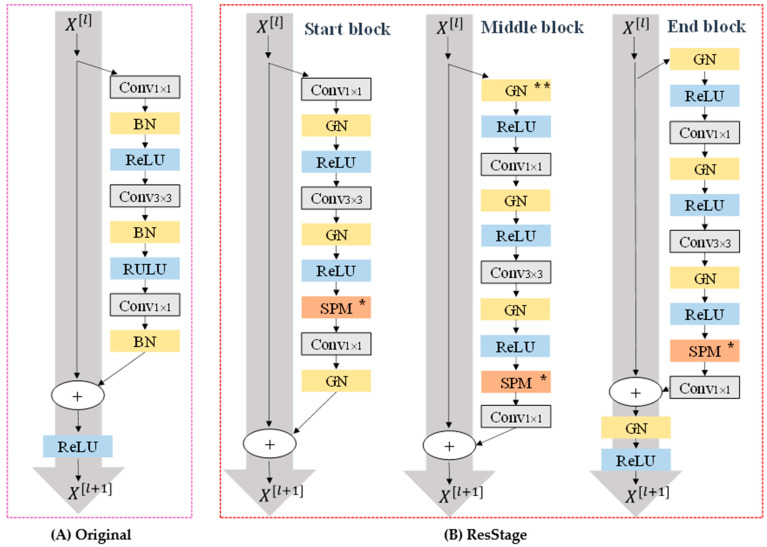
Improved ResNet block structure (**A**) original, (**B**) the structure of IResNet (** means the first GN in the first Middle ResBlock is eliminated in each stage, * means adding the module when channels of the feature map are 512).

In the actual experimental procedure, the information in the data is limited because of the specificity of the small sample dataset. After the training period, without learning new features, it is easy to overfit. It was found that, as shown in Figure 4A, ResNet first reduces the number of channels by 1 × 1 convolution layer, then captures features using a 3 × 3 convolution layer, and finally restores the number of channels through a 1 × 1 convolution layer. ReLU operation directly shields all negative signals on the straight connected road. In the small sample training period, we produce more negative signals and directly remove those negative signals, leading to the huge loss of information. In the case of small samples, data training is not worthwhile. Therefore, we have designed an effective way to communicate information, drawing on the idea of improving the residual network [29], so that the network cannot lose too much useful information in vain during the training of small sample data, and to avoid over-fitting the network.

We divide signal transfer in the ResBlock into three stages, where different stages mean that convolutional normalization will be performed in a different order, and the end of each stage would ensure nonlinearity, in every layer. These stages of change are determined by the size of space and the number of channels of the network output. As shown in Figure 4B, the ReLU operation on the straight connected channel is eliminated in the Start block phase. At the start of the next phase, ReLU is used for signal activation before the signal transmission continues. In the final stage, GN and ReLU are added to the straight connected channel to ensure the overall normalization and activation of the signal. Such a phased signal transmission, in essence, does not increase the complexity of the network but rather transmits the signal better. Later in the discussion section, we demonstrate this experimentally.

The SPM module is added to establish a self-attention mechanism for the feature target capture (when the number of network output channels is 512 and used). We used the group norm [30] to normalize the feature graph, because the normalization effect of batch normalization with a small batch size is poor, and the small sample dataset we trained is not suitable for using a large batch size, which may exacerbate the possibility of over-fitting. The group norm is a way to avoid this by naturalizing in the form of channel grouping. The effect of normalization depends on the number of groups and not the size of the batch. For the selection of the number of groups, we refer to the original paper [30] and choose the group with the best experimental effect in the paper.

## 4. Experiments and Results

### 4.1. Experimental Details

#### 4.1.1. Accuracy Assessment

We mainly used two methods to evaluate the accuracy of our model. However, in order to more vividly highlight the advantages and disadvantages of different algorithms in the application field of this paper (vacancy segmentation), we propose the better error calculation method, which will be mentioned later. The first is the widely used pixel accuracy (PA, which is calculated by dividing the number of truly predicted pixels by the total number of pixels), followed by the mean cross over union (mIoU). The mIoU formula used is:

(3) $$mIOU=\frac{1}{m}\overset{m}{\underset{i=1}{\sum }}|A_{i}\cap B_{i}\left|/\right|A_{i}\cup B_{i}|$$

where $A_{i}$ and $B_{i}$ indicate the area of class i in label and the area of class i in result mapping, respectively, and m indicates the number of classes.

These two methods are most commonly used for evaluating semantic segmentation models and naturally, both evaluation models are also used during our training. Given the particularity of our application scene, we also propose a more intuitive way to evaluate the effects of the actual application of the model, and we define the formula as follows:

(4) $$VR=\frac{V}{C+V}$$

where *V* represents vacant area, *C* represents crop area, and *VR* stands for vacancy ratio. Essentially, we use the vacancy ratio to measure the vacancy between the result of model’s segmentation of farmland and the actual manual segmentation result.

Based on this vacancy ratio, we further constructed the error calculation formula for the objective evaluation of the model effect as follows:

(5) $$error=\frac{\left|VR_{f}-VR_{p}\right|}{VR_{f}}$$

where $VR_{f}$ represents the actual vacancy rate, $VR_{p}$ represents the predicted vacancy rate, and the error value is obtained by dividing the absolute value of the difference by the actual vacancy rate. To distinguish the predictive effect when farmland is in a different state of vacancy, direct subtraction method is not chosen. For example, when there are fewer farm vacancies, such as between 1% and 2%, the fault tolerance of the predicted vacancy will become lower, because it is directly related to the visual effect. Fault tolerance becomes higher when the actual vacancy rate is already high, such as between 5% and 10%.

#### 4.1.2. Training Parameter Setting

Due to the large size of the training images, we use the NVIDIA Tesla V100 32GB to ensure normal training and experimental comparison of data. We selected cross-entropy loss as a loss function to measure the error between prediction result and manual standard. Due to particularity of the dataset, there are only three classes, and there is a huge vacancy between categories. Hence, we specially designed weights for each category. By analyzing the quantitative relationship between categories, we designed three weights, and finally, the experiment proved that weight = [0.8, 1, 1.2] has the best effect. The Loss formula is defined as follows:

(6) $$loss\left(x,class\right)=weight\left[class\right]\left(-x\left[class\right]+\log\left(\underset{j}{\sum }exp\left(x\left[j\right]\right)\right)\right)$$

where class represents three categories of different labels in the experiment. 0 represents the background class, 1 represents the crop category, and 2 represents the vacancy category, which is not directly involved in the calculation but acts as an index. Further, x represents the input vector, and each value in the vector represents the probability prediction value corresponding to each category.

For the optimizer settings, we chose Adam, the adaptive moment estimation, which is essentially the drive of RMSprop [31], to ensure that it learns when to adjust the parameters during training process, while achieving faster speeds and smaller parameters. The base learning rate is set at 0.01. In the training process, 10% random rotation, random horizontal and vertical rotation, and Gaussian noise are adopted. Details on basic tasks can be found in reference [32].

### 4.2. Results

**Figure 5:**
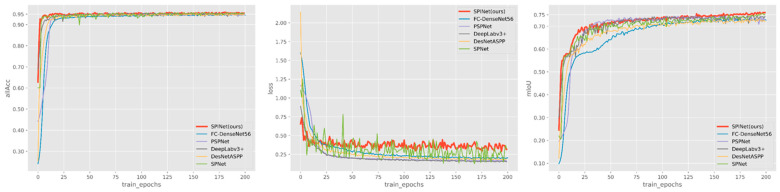
Accuracy, loss value, and mIoU of training process.

In the methods described above, we have a comparative analysis of each method, with the main training results as Table 1. From the table, it can be easily seen that the SPINet network based on encoder–decoder architecture in the test set achieved the best results in both accuracy and mIoU indicators, with an increase of nearly 4% points on the original basis and the lowest error rate of 7%. We then analyzed the results of the various indicators of the training corresponding to the different models in detail. From Figure 5, we can see that SPINet trains the fastest and quickly reaches higher accuracy. As can be seen from the training process of mIoU in Figure 5, compared to SPNet, the mIoU value in the training process of our proposed method becomes less volatile, smoother, and reaches a higher value. We mention our loss calculation in Part III. It can be found from the loss training curve, shown in Figure 5, that the loss value of SPINet is higher than that of other models, which indicates the addition of double loss so that the loss value does not fall rapidly to a lower value. The model can continue to learn more new features.

**Figure 6:**
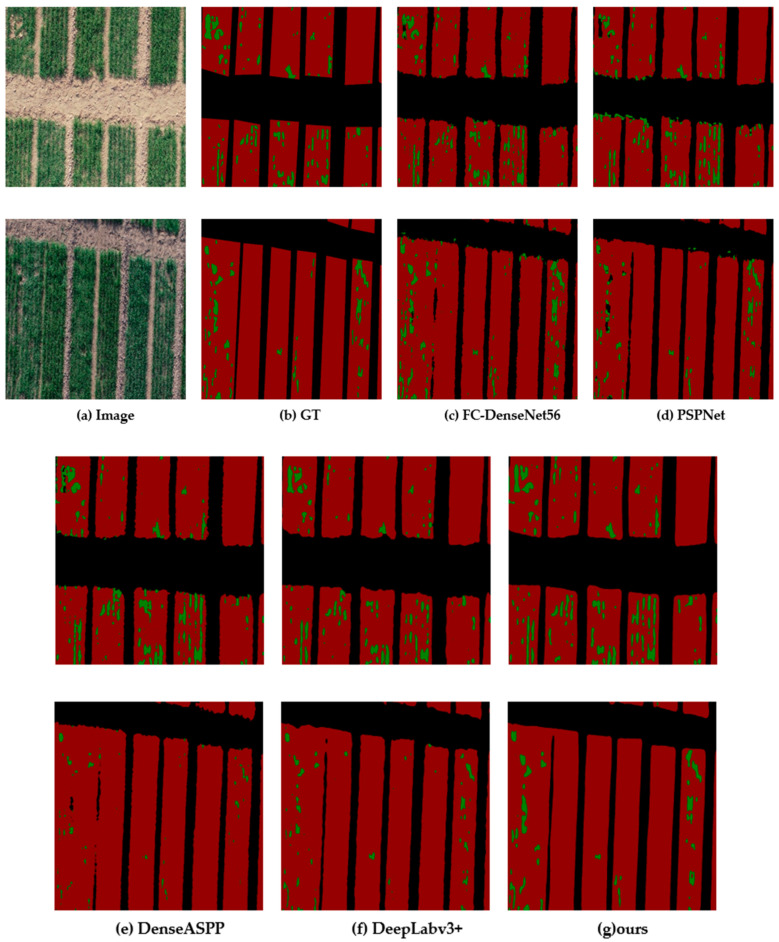
Segmentation results of different models. (**a**) Original image, (**b**) ground truth, and (**c**) visual results of FC-DenseNet56, and (**d**) for the visual results of PSPNet, (**e**) visual results of DenseASPP, (**f**) visual results of DeepLabv3 plus, and (**g**) visual results of our proposed.

In order to better display the comparative results of the models, we took out two pictures with obvious characteristics for analysis. Figure 6 shows the segmentation results of these two images by different models, which will be analyzed one by one later.

In Figure 6, one of the two images have a large vacancy, and the other one has many continuous strip vacancies. From the results of various models, it can be seen that the prediction of FC-DenseNet56 on the vacancy is incomplete, and there is a principal error; that is, the predicted vacancy position is outside the crop region and appears very messy. The prediction results of PSPNet are similar to those of FC-DenseNet56, precisely the rough edges. DenseASPP has largely improved the first two effects, but it still has flaws in its prediction of a big vacancy. DeepLabv3+ has had the most dramatic improvement because of a decoder, which is better at predicting edges and large vacancies. The comparison with SPNet model will be discussed in detail in the discussion section. Finally, the predicted effect of SPINet model is very close to the ground truth. Due to the use of strip pooling, which facilitates the capture of characteristics of vacancies, the segmentation of vacancies is very accurate, and the visualization effect is also very good.

## 5. Discussion

Vacancy segmentation is a branch of semantic segmentation and has a huge potential for its application in image crack detection (e.g., road crack detection). Farmland vacancies are handled in a different way than in previous datasets because of their relatively large randomness and irregularity. In this paper, a method of semantic segmentation of farmland vacancies is proposed, which is more accurate than several existing typical semantic segmentation methods. Based on this, our discussion is as follows:

### 5.1. Contribution to Cracks Segmentation

The difference between crack segmentation and general semantic segmentation application is that the image of crack segmentation has subtle features and small targets, making the segmentation method unsuitable for all kinds of traffic and character scenes. The segmentation model, described in this paper, based on farmland vacancy, can better solve this problem. Firstly, we analyze the differences and relation between vacancy segmentation and cracks segmentation. Although farmland vacancy and crack both belong to vacancy segmentation, it is obvious that farmland vacancy has more randomness in shape and size and is denser in distribution than ordinary cracks, which can also be confirmed from the actual images. Therefore, algorithms suitable for simple crack segmentation cannot be well applied in this field. Compared with other methods, the method proposed by us has proved by experiments that all indices have been significantly improved, especially by nearly 4% on mIoU. This method provides an idea for complex cracks segmentation and can be directly transferred to the field for use.

### 5.2. Contribution to Estimate of Crop Growth and Yield

As mentioned in the Introduction section, the growth process of crops is accompanied by considerable randomness, and all stages from germination to seedling to maturity may be affected by various environmental factors. Therefore, in order to analyze and understand the crop growth situation in time, scientific researchers have put forward many methods. Huilin Tao, Liangji Xu et al. [33], using a hyperspectral sensor installed on an Unmanned Aerial Vehicle (UAV), obtained vegetation index and red edge parameters and their combination. Using stepwise regression (SWR) the partial least squares regression (PLSR) method, above ground biomass (AGB), and the leaf area index (LAI), two kinds of plant growth parameters were estimated. In addition, Thomas Moeckel, Supriya Dayananda et al. [34] used UAV to collect images for 3D point cloud analysis of crop phenotypes. Yi Ma, Shenghui Fang et al. [35] used hyperspectral data to collect dry AGB, because it is an important parameter in assessing crop growth and predicting the yield. Vacancy ratios are not only meaningful in long-term estimation, but also an important indicator in crop growth analysis. At the same time, the segmentation of crops can be derived from the actual planting area of crops in the area, so that it can be directly applied to the crop yield estimate and also combined with the long-potential estimate and other data to carry out the yield estimate.

From the macro point of view, dividing crops and vacancies directly to obtain crop planting vacancy ratio can obtain not only the theoretical crop cover area, but also the actual crop area, which is a direct, effective, and simple way to estimate crop growth. It can be seen from our research that vacancy rate is important in growth estimation and is an important indicator in crop growth analysis. At the same time, the segmentation of crops in this paper can obtain the actual planting area of crops in this area. It can thus be directly used in crop yield estimation or combined with growth prediction and other data for yield estimation.

### 5.3. Different from Existing Methods

Based on the thin, long, and narrow characteristics of most cracks and vacancies, our model adopts the strip pooling idea of deploying a narrow kernel shape along a spatial dimension, to capture long-distance distributions of isolated areas. This is also, in fact, conducive to capturing the characteristics of cracks. At the same time, integration of the spatial pyramid module allows the model to detect effectively and split when other shapes appear in the crack. However, because the crack edge information is too low, in the normal upper sampling process, it is easy to lose its original information.

#### 5.3.1. Effects of Strip Pooling and Decoder

In order to verify the actual effect of the improved method proposed by us, we conducted detailed comparative experiments, and the results are presented in Table 2. SPNet101 is kept constant here. With the improved ResNet as backbone, mIoU increased by about 1% in the validation set. If only the decoder layer is added, the original SPNet101 as the encoder layer increases in accuracy by approximately 0.6% and mIoU by about 2%. When both are added, the accuracy improvement is small compared to just adding the decoder, but mIoU increases by nearly 1%. Hence, we believe that our improvements are effective, especially in the pixel recovery of feature maps, which can accurately restore the information on the vacancy edge of the crop and correctly split out the larger vacancies in the crop.

**Figure 7:**
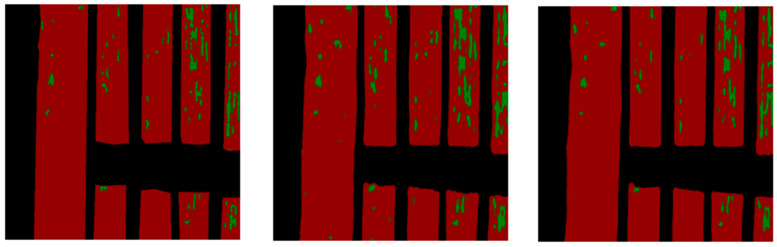
The effect of the decoder: From left to right- the ground truth, the segmentation result of SPNet101, and the segmentation result of the method we proposed.

In order to better illustrate this point, we chose an actual picture. From the original picture in Figure 7, it can be found that there are many vacancy distributions in crops, and part of the vacancy is long, in the shape of strips. Therefore, in the segmentation results of SPNet101 model, these vacancies can be well segmented. However, compared with ground truth, its excessive ability to predict vacancy leads to more wrong categories that should not be predicted. In comparison, the model proposed by us achieves a balance in this process, with neither excessive prediction nor little prediction, the prediction result is very similar to the ground truth.

#### 5.3.2. Comparison with Other Methods

During the experiment, we compared the application of FC-DenseNet56, PSPNet, and other methods in the dataset and obtained the experimental results of each method. In this section, we discuss each method along with the experimental results and the experimental process.

Here, we will macroscopically analyze the parameters of different methods used in this paper. As we all know, DenseNet structure benefits from the design of a dense block, which makes the network narrower (that is, fewer channels) and reduces parameters to a certain extent. The parameters of the two models (FC-DenseNet56 and DenseASPP) derived from this are relatively small. However, as we will discuss later, the DenseNet network is not suitable for this application field, especially as there are large defects in the visualization results. In contrast, PSPNet has increased parameters on the basis of FCN, but the application effect is not good. DeepLabv3+ increases the number of parameters in a series of improvements, but it designs the depthwise separable convolution, which drastically reduces computation complexity, and its application effect is also worthy of praise. However, it is worth noting that the number of additional parameters added in SPM and MPM in this paper is only about half of that of the pyramid pooling module (PSPNet). Moreover, the increase of parameters brought by a simple and effective decoder is almost negligible. At the same time, the information transmission mode is improved for ResNet, but the number of parameters is not increased. Therefore, by comprehensive comparison, the model achieves good results under the condition of limited parameter increase.

First of all, with DenseNet as the backbone, we found that its features of dense jump connections are not suitable for applications in this field. In this field, only two categories need to be separated, crops and vacancies, where crops are the big targets and vacancies are the small ones. Multi-scale fusion and feature reuse in DenseNet grasps the characteristics of crop categories but does not distinguish between the vacant part of crops during subsampling, which leads to negligence of vacant part in the process of upsampling and image recovery. FC-DenseNet56’s fully connected approach mitigates this shortcoming, but makes the prediction of vacancies very messy and learning less effective. As DenseNet, the backbone of DenseASPP, can generate a very large range of receiver field features by using atrous spatial pyramid pooling (ASPP), due to the particularity of the vacancy features, can lose more information in the process of final upsampling and recovery. Similarly, for PSPNet, in the training process, we found that the spatial pyramid pooling network is not good at vacancy segmentation, and the prediction of a big vacancy is often wrong in the background. Instead, DeepLabV3 constructed with an encoder–decoder structure shows better adaptability in this dataset and obtains a better model with faster training effect. It also uses multiple sampling rates to expand convolution. The encoder–decoder structure captures clear target boundaries by gradually restoring spatial information. In these training processes, we have summarized the following points, which are the direct sources of our improvement:

- The limited amount of information learned in the early stages of the backbone model, as a model of ResNet, leads us to wonder if much information was lost during the original transmission of ResNet.
- The encoder–decoder structure recovers the target boundary information satisfactorily, especially the prediction accuracy of the vacant edge. This prompts us to think about building a network model of an overall encoder–decoder architecture.
- As a single loss equation is used for all the previous training processes, training in small samples of those methods will quickly drop to a relatively low value, and the ability to learn new features of pictures will significantly reduce. In this regard, we use the method of loss reuse to improve this effect. The value of loss in Layer1 is added to the final calculation of loss with a certain proportion so that the value of loss will not be reduced to the point that it cannot be learned, but will be repeatedly learned in the process of oscillation. This is also determined by the characteristics of small-sample training.

### 5.4. Limitations and Future Work

The manual labeling of the vacancy segmentation of farmland images is very tedious and requires a lot of effort, which is also the reason why we chose the small sample training network. Moreover, it is difficult for the naked eye to distinguish between the vacant categories that are not obvious, making the results obtained by training and manually marked results erroneous. Such errors are not due to the model itself, but due to the fact that the two measures of vacancy are not exactly the same (mainly for a small vacancy). Therefore, we also propose an evaluation method that can better reflect the effect of the model to alleviate this problem. Additionally, this study is aimed at the training of a small sample of farmland in a certain region. Although the training speed is fast and the training effect is good for particular farmland, to be applied to other farmlands, their information needs to be collected for further training to achieve generalization. Therefore, in the future, farmland data of different regions and different terrains will be collected, and further enhancement in the adaptability and application range of the model through data augmentation training will be made.

Secondly, this paper considers the improvement of prediction accuracy and analyzes the problem of increasing parameters macroscopically, but does not calculate it in detail. In the future, more detailed studies will be made on this point to optimize the algorithm to reduce the number of parameters as much as possible.

## 6. Conclusions

Earlier, satellite image or aerial image labeling was directly used for farmland segmentation. However, it was too complicated for farmland segmentation in small areas and could not directly highlight the crop growth situation of farmland in such areas. Direct and rapid observation of crop growth in a certain region plays a significant role in rational planting density, crop growth prediction, crop growth analysis, and other aspects. Hence, we establish a semantic segmentation module based on encoder–decoder architecture, where SPINet as an encoder can automatically extract in-depth crop and vacancy information, set-up the decoder to recover extracted feature information, realize end-to-end training and prediction, and achieve good segmentation effect. Thus, from our experiments and comparative analysis, we draw the following conclusions:

Firstly, we construct a dataset of field crop and vacancy segmentation, which provides a new idea for studying field crop segmentation.

Secondly, the application effect of strip pooling in vacancy segmentation is very significant, which can adaptively capture the useful information of vacancy and then conduct accurate segmentation. Meanwhile, the encoder–decoder network structure is very effective for boundary pixel recovery of segmented targets.

Finally, the farmland segmentation method proposed in this paper reached 95.6%-pixel accuracy, and the mIoU value reached 77.6%, reaching the level of practical application. This method can be applied to crop growth analysis, assessment of crop growth, yield estimation, and other practical fields.

## Figures and Tables

**Table 1:** Results of several methods on the test set, where error is the result calculated from the formula above.

Methods	Accuracy (%)	mIoU (%)	Error
FC-Densenet56 [16]	94.2%	73.3%	32%
PSPNet [17]	94.3%	73.0%	23%
DenseASPP [18]	93.3%	69.3%	46%
DeepLabv3+ [22]	95.0%	73.8%	24%
SPNet [32]	95.1%	75.5%	19%
SPINet (Ours)	**95.6%**	**77.6%**	**7%**

**Table 2:** Impact of decoder addition and use of IResNet on results of validation set.

Setting	IResNet	Decoder	Pixel Acc. (%)	mIoU (%)
SPNet-101			94.7%	75.8%
SPNet-101	**√**		94.5%	76.4%
SPNet-101		**√**	95.3%	78.8%
SPNet-101	**√**	**√**	**95.6%**	**79.8%**

## Data Availability

The following are available online at: https://drive.google.com/drive/folders/1tMsFK1swDTPZt_0Np14B_PAPFM7GsXJB?usp=sharing (accessed on 10 March 2021). The dataset of farmland used in the experiment can be obtained here. As we are still doing more research on the dataset, we will upload our dataset to the same sharing link later.
